# Role of Chemokine (C–X–C Motif) Ligand 10 (CXCL10) in Renal Diseases

**DOI:** 10.1155/2020/6194864

**Published:** 2020-02-05

**Authors:** Jie Gao, Lingling Wu, Siyang Wang, Xiangmei Chen

**Affiliations:** ^1^Department of Nephrology, Chinese PLA General Hospital, Chinese PLA Institute of Nephrology, Beijing Key Laboratory of Kidney Disease, State Key Laboratory of Kidney Diseases, National Clinical Research Center for Kidney Diseases, Fuxing Road 28, Beijing 100853, China; ^2^Department of Nephrology, Shandong Provincial Hospital Affiliated to Shandong University, Jingwu Road 324, Jinan 250000, China

## Abstract

Chemokine C–X–C ligand 10 (CXCL10), also known as interferon-*γ*-inducible protein 10 (IP-10), exerts biological function mainly through binding to its specific receptor, CXCR3. Studies have shown that renal resident mesangial cells, renal tubular epithelial cells, podocytes, endothelial cells, and infiltrating inflammatory cells express CXCL10 and CXCR3 under inflammatory conditions. In the last few years, strong experimental and clinical evidence has indicated that CXCL10 is involved in the development of renal diseases through the chemoattraction of inflammatory cells and facilitation of cell growth and angiostatic effects. In addition, CXCL10 has been shown to be a significant biomarker of disease severity, and it can be used as a prognostic indicator for a variety of renal diseases, such as renal allograft dysfunction and lupus nephritis. In this review, we summarize the structures and biological functions of CXCL10 and CXCR3, focusing on the important role of CXCL10 in the pathogenesis of kidney disease, and provide a theoretical basis for CXCL10 as a potential biomarker and therapeutic target in human kidney disease.

## 1. Introduction

Kidney disease is a major public health burden that affects the quality of life of millions of people. Renal dysfunction can result from or cause a variety of pathologies. Inflammation and immune system activation have been considered common underlying mechanisms [1]. Chemokines are a large family of small cytokines with a molecular weight between 7 and 15 kDa [2]. Chemokines and their receptors play critical roles in the recruitment, activation and adhesion of various types of leukocytes to inflammatory foci [3]. The subgroups of chemokines, C, CC, CX3C, and CXC, are defined based on the configuration of a conserved aminoproximal cysteine-containing motif. With the exception of the C subgroup, all chemokines contain a common four-cysteine residue motif linked by disulfide bonds in conserved positions, one between the first and third cysteines and one between the second and fourth cysteines, to form triple-stranded b-sheet structures [4]. As a general rule, C chemokines mainly recruit lymphocytes, and CC chemokines are chemotactic factors for monocytes [5]. CX3CL1 (fractalkine), the only CX3C chemokine, combines the properties of chemoattractant (T cells and monocytes) and adhesion molecules [6]. CXC chemokines, on the other hand, are further classified according to the presence of the tripeptide motif glutamic acid-leucine-arginine (ELR) in the N-terminal region. ELR^+^ CXC chemokines mediate neutrophil attraction and angiogenesis, typically via interaction with chemokine (C–X–C motif) receptor 2 (CXCR2), whereas ELR^−^ CXC chemokines attract lymphocytes and have angiostatic properties through their common receptor CXCR3 [5, 7]. CXCL10 is an ELR^−^ CXC chemokine also known as interferon-inducible protein-10 (IP-10). In this review, the role of CXCL10 in different renal disease models will be highlighted. Under the influence of a proinflammatory cytokine milieu, CXCL10 can be secreted by leucocytes and tissue resident cells and plays a significant role in the recruitment of immune cells expressing CXCR3. In addition to its role in chemotaxis, CXCL10 also participates in regulating cell growth and inhibiting angiogenesis during tissue injury, development, and maintenance. Recent reports have shown that CXCL10 is involved in the pathological processes of diverse human kidney diseases, such as mesangial proliferative glomerulonephritis (MesPGN), acute kidney injury (AKI), and nephrotoxic nephritis. Furthermore, CXCL10 has been identified as a major biological indicator of disease severity and may be utilized as a prognostic indicator for diseases such as renal allograft dysfunction and lupus nephritis (LN). The aim of this review is to summarize the current understanding of the role of CXCL10 in various renal diseases, mainly focusing on the mechanism elucidated in both experimental and clinical studies.

## 2. Chemokine CXCL10 and Its Receptor CXCR3

CXCL10 is also named IP-10. The human Cxcl10 gene was initially isolated in 1985 by Luster et al. [8] after treating a lymphoma cell line (U937) with recombinant interferon- (IFN-) *γ*. Many cytokines may regulate the cellular expression of the Cxcl10 gene. CXCL10 is strongly induced by IFN-*γ* and by the type I IFNs IFN-*α*/*β* and weakly induced by tumor necrosis factor, although tumor necrosis factor synergizes strongly with IFNs to induce CXCL10 [9]. Not surprisingly, the induction of CXCL10 occurs almost universally in humans during the course of cell-mediated immune responses evoked in a variety of pathologic states, including infection, allograft rejection, and autoimmunity [10]. Under inflammatory conditions, type-1 helper (Th1) activation induces IFN-*γ* and TNF-*α* production, which stimulates CXCL10 secretion by lymphocytes and by other cells, such as neutrophils, monocytes, endothelial cells, fibroblasts, thyrocytes, and keratinocytes [11]; this process creates a positive feedback loop that initiates and perpetuates the immune cascade [12].

CXCL10, as well as two other structurally related IFN-inducible C–X–C chemokines CXCL9 (monokine induced by gamma IFN) and CXCL11 (IFN-inducible T cell alpha chemoattractant), exerts its biological effects mainly via binding to CXCR3, a seven transmembrane spanning G protein-coupled receptor (GPCR), that is mainly expressed on Th1 CD4^+^ T cells, effector CD8^+^ T cells, and innate-type lymphocytes, such as natural killer (NK) and NKT cells [13]. CXCR3 is also expressed on the surface of stromal cells (i.e., endothelial cells, renal mesangial cells, trophoblasts, and keratinocytes) [14].

Three splice variants of CXCR3 resulting from alternative splicing of three different exons have been identified: CXCR3-A, CXCR3-B, and CXCR3-alt. These variants are differentially expressed in different cell types, resulting in cell type-dependent effects. CXCR3-A is the main isoform and is expressed in most cell types and codes for a protein of 368 amino acids and couples with G*α*i to activate the ERK1/2, p38/MAPK, JNK, and PI3-kinase/Akt signaling pathways. CXCR3-A mainly induces intracellular calcium influx, DNA synthesis, and cell proliferation and preferentially mediates Th1 cell chemotaxis [15–17]. CXCR3-B codes for a larger protein of 416 amino acids, couples with G*α*s to activate adenylyl cyclase, and inhibits endothelial cell proliferation and migration [16, 18, 19]. CXCR3-alt, which is known to be coexpressed with CXCR3-A at a very low level, codes for a truncated receptor of 260 amino acids. The biological role of CXCR3-alt is still unknown [15, 20, 21]. Signaling through these alternatively spliced isoforms of CXCR3 appears to be the key mechanism by which CXCL10 exerts its versatile biological functions on different cell types. All three isoforms of CXCR3 have been identified in humans, but Lu et al. [22] found that murine Cxcr3 gene did not encode CXCR3-B splicing variant, only one isoform of CXCR3, similar to human CXCR3-A, has been identified in mice [5].

CXCL10 can also function via CXCR3-independent pathways in vivo and in vitro. CXCL10 can bind to glycosaminoglycans (GAGs) and is involved in inhibiting endothelial cell proliferation [23] and fibroblast migration [24] independent of CXCR3 signaling. The antifibrotic effects of CXCL10 in myocardial infarction are independent of CXCR3 and may be mediated through proteoglycan signaling [25]. In addition, CXCL10 induces *β* cell apoptosis by binding to TLR4 in pancreatic *β* cells [26], and the TLR4–IRF3 pathway is involved in regulating CXCL10 expression in the Habu nephritis model [27]. Interestingly, there is evidence for CXCL10 signaling independent of binding to CXCR3 or GAGs that might be related to epithelial and endothelial cell function, but the exact mechanism has not yet been described [5, 28].

## 3. The Expression of CXCL10 and CXCR3 in Resident Renal Cells

Previous studies have found that CXCL10 can be released by renal cells. It has been reported that CXCL10 can be expressed by mesangial cells, tubular epithelial cells, podocytes, and endothelial cells after stimulation in a cell type- and stimulus-specific manner.

CXCL10 and CXCR3 are expressed at low levels in the normal kidney, but their expression levels are upregulated under pathological conditions. Immunostaining of CXCL10 in rats was observed by Han et al. in a linear pattern along the glomerular capillary loop, and CXCR3 staining coincided with the pattern of epithelial cells along the glomerular capillary wall [29]. Romagnani et al. also showed by immunostaining that CXCR3 was mainly expressed in the afferent arterioles in the normal human kidney [30].

### 3.1. Mesangial Cells

In 1993, IFN-*γ*, LPS, and other agonists were shown to increase CXCL10 expression in cultured murine mesangial cells [31]. In addition, human mesangial cells stimulated with soluble IgA aggregates (AIgA) showed a time- and dose-dependent increase in CXCL10 mRNA expression [32]. Romagnani et al. found high CXCL10 mRNA and protein expression levels by using in situ hybridization and immunohistochemical analyses in kidney biopsy specimens from patients with glomerulonephritis (GN), particularly from those with membranoproliferative GN (MPGN). The induction of CXCL10 mRNA and protein expression by IFN-*γ* or IFN-*γ* plus TNF-*α* was also detected [33].

Chemokine receptor CXCR3 expression on human mesangial cells was also published by Romagnani and colleagues [30]. They found that CXCR3 was highly expressed in glomerular structures and localized to mesangial cells of patients with proliferative glomerulonephritis (PGN), as demonstrated by morphology and double-label immunohistochemistry with monoclonal antibodies (mAbs) against CXCR3 and *α*-SMA, a disease marker of mesangial cells. CXCR3 was also detected by flow cytometry in cultured human mesangial cells, in which it appeared to be functionally active. It was also showed that the expression of CXCR3 was wildly found in ureteric buds, comma, and S-shaped bodies in developing kidneys and increased gradually with the development of the kidney. Double labeling immunohistochemical analysis of the CXCR3 and *α*-SMA, the specific marker of mesangial cells, confirmed that CXCR3 was expressed in the developing mesangium [34].

However, another study reported that CXCR3-positive cells were rare in glomerular tufts but were a major constituent of tubulointerstitial infiltrate. Segerer et al. [35] studied CXCR3-positive cells in renal biopsies from patients with GN by immunohistochemistry and found that CXCR3-positive cells were relatively rare in glomerular tufts, as there was no evidence of CXCR3 on mesangial cells, and the main site of CXCR3 expression was infiltrating T cells in the tubulointerstitium. Moreover, CXCR3 and CXCL10 mRNA expression was found in the tubulointerstitial compartment but was hardly detected in glomeruli, consistent with the immunohistological data.

The discrepant results of these two studies may stem from the use of different anti-CXCR3 antibodies. Romagnani et al. used the antibody 49801.111 (R&D Systems), whereas Segerer et al. chose clone 1C6 (BD Biosciences Pharmingen). As the antibody 49801.111 recognizes an epitope that is constitutively expressed on smooth muscle cells, the finding of increased immunoreactivity on glomerular mesangial cells during GN might not be surprising, as mesangial cells upregulate markers of smooth muscle cells during glomerular inflammation [35]. To further confirm this hypothesis, Segerer and colleagues investigated CXCR3 protein expression in human mesangial cells (HMCs) by FACS analysis with the two mAbs. Despite the absence of CXCR3 mRNA in cultured HMCs, a positive signal was found with clone 49801.111 (R&D Systems), whereas clone 1C6 (BD Biosciences Pharmingen) revealed no signal, even after cells were treated with proinflammatory cytokines. There is potentially an unknown variant of CXCR3 or another undefined chemokine receptor that mediates the functional responses of CXCL10 in mesangial cells, and this issue must be addressed in further studies [35].

### 3.2. Tubular Epithelial Cells

As previously reported, cultured tubular epithelial cells stimulated with IFN-*γ* and TNF-*α* express CXCL10 and CXCR3 at both the mRNA and protein levels [36]. Moreover, activation of renal tubular cells by infiltrating T cells induces CXCL10 production mediated by either soluble or cell contact-dependent factors, which may amplify and perpetuate local inflammation [37]. In addition to the in vitro evidence, there is in vivo evidence that tubular epithelial cells are positive for CXCL10. Urinary CXCL10 levels were significantly elevated in samples collected from recipients with acute tubular injury and acute renal allograft rejection [38]. This finding is consistent with reports that CXCL10 expression was significantly upregulated after ischemic-reperfusion injury [39] or unilateral ureteral obstruction [36] in mice.

### 3.3. Podocytes

A variety of studies have shown that CXCL10 and its receptor CXCR3 are expressed in podocytes. Huber et al. [40] reported that CXCR3 mRNA was expressed on human glomeruli and positive fluorescence staining for CXCR3 was detected in podocytes. CXCL10/CXCR3 expression was analyzed in two experimental models of nephrotic syndrome, puromycin aminonucleoside nephropathy (PAN) and anti-nephrin antibody-induced nephropathy (ANA), both with slit diaphragm (SD) dysfunction resulting in proteinuria [41, 42]. CXCL10/CXCR3 mRNA expression was increased in both PAN and ANA, and CXCL10/CXCR3 immunostaining was greater in glomeruli at the peak of proteinuria. Another group observed noticeable CXCL10 expression in a linear-like pattern in rat glomeruli in anti-Thy1.1 antibody-induced GN (Thy 1.1 GN) [29]. In addition, double-staining immunofluorescence (IF) studies showed that elevated CXCL10 expression occurred mainly at the podocyte (clone 4D5). In vitro, CXCL10 and CXCR3 expression was clearly detected in differentiated conditioned podocytes with IF, RT-PCR, and Western blot. Recent studies reported that stimulation of cultured podocytes with both IFN-*γ* and TNF-*α* markedly induced Cxcl10 mRNA expression and CXCL10 secretion [42].

### 3.4. Endothelial Cells

In vitro assays have revealed that CXCL10 can be secreted by glomerular endothelial cells (GEnCs) [43, 44]. Panzer et al. reported that renal CXCL10 mRNA and protein expression were markedly upregulated in a model of renal endothelial microvascular injury (REMI). To identify the cellular sources of intrarenal CXCL10 mRNA expression, in situ hybridization was performed, and the results revealed that CXCL10 mRNA was selectively expressed by endothelial cells in the tubulointerstitial area colocalizing with infiltrating T cells. Despite extensive damage of the glomerular vasculature, no CXCL10 expression by GEnCs was detected [45]. In addition, double-label immunohistochemical analyses of developing kidneys showed colocalization of CXCL10 with peritubular capillary endothelial cells (endothelial antigen CD31, [34]).

## 4. CXCL10 and Renal Diseases

### 4.1. Mesangial Proliferative Glomerulonephritis (MesPGN)

MesPGN is a common kidney disease that includes IgA nephropathy (IgAN) and non-IgA MesPGN [46, 47]. MesPGN is an autoimmune inflammatory disease and is characterized by the proliferation of resident mesangial cells and the expansion of the mesangial matrix [48–50]. Recent studies have expanded the understanding of chemokine function in the detailed molecular mechanisms of GN [51, 52].

In the last few years, a strong experimental and clinical evidence has been obtained indicating that CXCL10 not only is responsible for renal inflammation by attracting T cells with a predominant Th1 profile [53] but also can directly contribute to mesangial cell proliferation. In 1999, Romagnani et al. [30] first reported high CXCR3 expression in mesangial cells from patients with PGN, such as IgA nephropathy, MPGN, and rapidly progressive GN. Moreover, CXCR3 was also observed on the surface of cultured HMCs and seemed to mediate both intracellular Ca^2+^ influx and cell proliferation. Furthermore, high CXCL10 mRNA and protein expression levels were detected in kidney biopsy specimens from patients with GN by in situ hybridization and immunohistochemical analyses. Double-label immunohistochemical analysis for proliferating cell nuclear antigen (PCNA) and *α*-SMA, which identifies proliferating mesangial cells, demonstrated that CXCL10 synthesis by resident glomerular cells was an extremely selective property of kidneys exhibiting PGN, in agreement with its proposed roles in regulating mesangial cell survival, chemotaxis, and proliferation [33]. In addition, CXCL10 was shown to induce HMC proliferation in a dose-dependent manner [54]. To further investigate the proliferation effect of CXCL10 in MesPGN, our previous study used Cxcl10-deficient mice (Cxcl10^−/−^ mice) to generate a Habu nephritis model, a classical experimental model that develops glomerular lesions similar to those of human MesPGN, and found that Cxcl10^−/−^ mice exhibited mitigated proliferation with less extracellular matrix accumulation and fewer cells in the glomeruli compared with wild-type mice in the mesangial proliferation period, which peaked on day 7 after HSV injection. In addition, we hypothesized that CXCL10 modulates cell cycle regulatory proteins to promote mesangial proliferation via the ERK signaling pathway, as confirmed in vitro using primary mouse mesangial cells [55]. Our data, together with those reported previously by Romagnani et al., were consistent with a cell type-specific proliferative effect of CXCL10 and suggested the potential therapeutic target of CXCL10 on cell proliferation.

In contrast with above findings, our colleagues Wu et al. [27] proposed that CXCL10 expression induced by Mxi1 inactivation leads to mesangial cell apoptosis in murine Habu nephritis. They used Mxi1-deficient mice to generate a Habu nephritis model and found a high CXCL10 expression in the mesangial dissolution period, which promoted apoptosis by activating caspase 3. Moreover, CXCL10 expression was induced by TLR4–IRF3 signaling in Mxi1-inactivated mouse mesangial cells (MMCs). In addition, Vlahakis et al. [56] proposed that G protein-coupled chemokine receptors can activate both survival and apoptotic signaling pathways. It has been reported that CXCL10 can trigger apoptosis in several cellular models, such as acinar cells [57], neurons [58], HeLa cells [59], T lymphocytes [60], beta cells [61], and microvascular endothelial cells [62]. Taken together, these observations suggest that the dominant role of CXCL10 varies in different disease stages and CXCL10 has the potential to exert opposite functions under different circumstances. As we observed, CXCL10 alone is a modest inducer of proliferation or apoptosis; such an observation is common in a complex pathologic process that involves several chemokines. It can be argued that the observed regulatory function might not correspond to a pure CXCL10 response; rather, some of the observed effects might stem from overlapping consequences of other chemokines and cytokines secreted under certain pathological conditions, and this clearly needs to be studied in more detail.

In addition to regulating cell growth, CXCL10 plays a pivotal role in maintaining podocyte function during Thy 1.1 GN, a classic, reversible model of MesPGN. Han et al. [29] reported that elevated CXCL10 expression during the development of Thy1.1 GN was detected mainly in podocytes, although some positive staining for CXCL10 was also detected in the mesangial area, but no CXCL10-positive cells costained with inflammatory cells. In addition, a blocking study with an anti-IP-10 mAb showed a decrease in the expression of podocyte-associated proteins such as nephrin and podocin that are essential for maintaining podocyte function, accompanied by exacerbated proteinuria, mesangiolysis, and matrix expansion. These findings indicate that exacerbated glomerular alterations caused by CXCL10 blockade do not result from the modulated inflammatory response but evidently act on podocytes. It was previously reported that podocyte dysfunction is intimately involved in the prognosis of mesangial alterations [63]. Shih et al. [64] reported podocyte dysfunction in the knockout mouse of CD2-associated protein, a component of the SD, accompanied by mesangial cell hyperplasia and extracellular matrix deposition. Han et al. hypothesized that the exacerbated proteinuria and mesangial alterations in Thy1.1 GN result from podocyte dysfunction caused by CXCL10 blockade.

Taken together, these observations may elucidate at least some of the mechanisms involved in the pathogenesis of MesPGN. It is reasonable to speculate that the initial event, such as the deposition of immune complexes, interactions of IgA with Fc receptors expressed on mesangial cells, or other insults, may induce CXCL10 production by resident glomerular cells. CXCL10 responds to pathological stimuli and selectively regulates the function of mesangial cells or podocytes. It is known that various cytokines are present during disease progression; an in vivo response mediated by CXCL10 alone in MesPGN is unlikely.

### 4.2. Acute Kidney Injury (AKI)

AKI is a multifactorial and multiphasic renal disease characterized by a rapid decline in renal function, resulting in the accumulation of metabolic waste and toxins, consequent complications, and the failure of other organs. Pathologically, AKI is characterized by renal tubular damage, inflammation, and vascular dysfunction [65, 66]. There is accumulating evidence that interstitial infiltrating leukocytes attracted and activated by chemokines are key mediators of the pathogenesis of tubular necrosis, the regeneration of the necrotic area, or interstitial fibrosis [66]. A growing body of evidence suggests that CXCL10 plays a role in AKI-induced inflammatory mechanisms by binding to CXCR3, which is predominantly expressed on activated Th1 cells.

A number of recent studies have demonstrated that CXCL10 expression is significantly elevated during AKI. Ho et al. [67] compared serial urinary proteomes of patients with (serum creatinine increase > 50%) or without AKI before, during, and after cardiopulmonary bypass surgery and found that CXCL10 was upregulated postoperatively in patients with AKI, which corresponds temporally to the later phase(s) of tubular injury unique to patients with AKI. Vaidya et al. [68] observed that the median urinary concentration of CXCL10 was significantly elevated in patients with clinically established AKI; this parameter performed well in differentiating patients with and without AKI. In addition, it has been reported that patients with influenza A/H1N1 infection and ARDS/AKI have overproduction of CXCL10 that possibly contributes to kidney injury and is associated with a higher risk of death [69]. Taken together, the data indicate that CXCL10 may be a good injury biomarker and has value in AKI diagnostics and prognostics.

Furthermore, upregulation of CXCL10 mRNA has been observed in mouse kidneys after ischemia and reperfusion (I/R). Fiorina et al. [70] observed the induction of CXCL10 and CXCR3 expression following kidney I/R injury and reported that CXCR3-deficient mice exhibited attenuated renal dysfunction compared with wild-type mice. This protective effect of CXCR3 deficiency was due to decreased recruitment of Th1 cells in the ischemic kidney. Recently, a nonpeptide chemokine antagonist (TAK) blocking both CCR5 and CXCR3 has been described, which could suppress the infiltration of T cells and NKT cells and contribute to tissue protection in renal I/R injury [71]. In addition to the potential role of CXCL10 and CXCR3 in regulating postischemic inflammation, Furuichi et al. [39] provided evidence that CXCL10 inhibits renal tubular cell proliferation in a mouse model of renal I/R injury. They found that administration of an anti-CXCL10 antibody increased the number of Ki67-positive tubular cells and mitotic tubular cells after I/R; the in vitro data are consistent with the in vivo data. Unexpectedly, no significant differences in inflammatory cell infiltration or the extent of tubular necrosis were found upon administration of functional blocking anti-CXCL10 antibodies. This finding may be related to incomplete inhibition by the antibody or a collaborative effect of CXCR3 ligands, including CXCL9/Mig and CXCL11/I-TAC. In addition, chemokine receptors other than CXCR3 on Th1 cells, such as CCR5 or CX3CR1, might participate in the migration of CXCR3-positive cells in I/R injury. Furthermore, as previously reported, lymphocytes account for only approximately 10% of total leukocytes in temporal renal ischemia injury [9, 39, 72].

Although necrotic area repair by tubular epithelial cells occurs after I/R injury, some parts of the injured kidney progress to interstitial fibrosis, a characteristic pathological change of chronic kidney disease [73]. Other putative functions of CXCL10 include the inhibition of fibroblast migration (anti-fibrotic) [74] and angiostatic effects. A recent investigation showed that CXCR3-deficient mice had impaired angiogenesis in a model of hindlimb ischemia, associated with decreased T cell and macrophage infiltration in the ischemic area [75]. However, the significance of these properties in the biology of tissue ischemia remains to be fully investigated [70, 76].

### 4.3. Lupus Nephritis (LN)

Systemic lupus erythematosus (SLE) is a chronic, potentially life-threatening disease characterized by a broad range of clinical manifestations, an often unpredictable temporal sequence of organ involvement, and disease flares that can cause permanent injury [77]. Although almost all organs in the body can be involved, LN is one of the most serious complications and can be seen in up to 60% of all SLE patients. As reported previously, CXCL10 expression is highly upregulated and correlates with disease activity in SLE patients [78]. Animal experiments suggested that CXCL10 expression is involved through CXCR3 in the pathogenesis of pulmonary inflammation in MRL/lpr mice associated with Th1 cell migration [79]. Recently, accumulated evidence suggests that CXCL10 and infiltrating CXCR3-positive cells may participate in the pathogenesis of LN.

Avihingsanon et al. detected urinary CXCL10 and CXCR3 mRNA levels in 26 patients, 14 of whom had class IV nephritis, and found that elevated urinary CXCL10 and CXCR3 levels could distinguish class IV LN from others with an accuracy greater than the current available clinical markers, namely, the SLE disease activity index (SLEDAI), proteinuria, renal function, or urinalysis. Furthermore, patients who responded to therapy had significantly lower CXCL10 levels, suggesting that CXCL10 can be used as a barometer of treatment efficacy [80]. Subsequently, Morimoto et al. used ELISAs to identify increased serum CXCL10 levels in patients with proliferative LN (World Health Organization (WHO) classes III and IV) [81]. Further research on the expression and pathological roles of the chemokines CXCL10 and CXCR3 in LN was conducted by Enghard and coauthors; they reported abundant expression of CXCR3 and CXCL10 in the interstitial infiltrate in renal biopsy tissues from 18 patients with LN, and the CXCR3-positive cells colocalized with CXCL10-producing cells. Furthermore, they found a selective enrichment of CXCR3^+^CD4^+^ T cells in the urine using flow cytometry, and CXCR3 expression on urinary T cells correlated with disease activity as determined by the SLEDAI, indicating that these biological molecules are promising biomarkers of acute LN [82]. Consistent data were obtained by Marie and colleagues: urinary excretion of CXCL10 was significantly higher in a cohort of LN versus non-LN patients and a statistically significant positive correlation was identified between CXCL10 and other variables, including urinary protein level, SLEDAI score, renal activity score, and grade of renal biopsy. CXCL10 was considered a highly valid predictor of SLE nephritis with high sensitivity (100%) and specificity (98%) [83].

However, there have been conflicting reports on the reliability of CXCL10 as a diagnostic tool for LN patients. Abujam et al. measured CXCL10 protein levels in serum and urine from 136 SLE patients, including 46 with active renal lupus. These authors concluded that urinary CXCL10 was indicative of renal activity but was not better than conventional markers such as C3, C4, and dsDNA in differentiating active renal from nonrenal SLE by ROC analysis [84]. Subsequently, El-Gohary et al. conducted a trial including 10 SLE patients with renal involvement (six active and four inactive). Although significantly increased serum and urinary CXCL10 levels were detected in active SLE patients, there was no difference between LN and SLE patients without renal involvement or between active renal SLE and active nonrenal SLE patients [85]. Furthermore, a cross-sectional cohort study indicated that urinary concentrations of CXCL10 could not distinguish active from nonactive renal disease in patients with juvenile-onset systemic lupus erythematosus (JSLE) [86]. These differences could be attributed to the relatively small sample size of some studies, differences in patient cohort characteristics, or differences in the utilized assay. However, urinary CXCL10 has promise as a potential biomarker for LN, but further investigation is required before it can be accepted as a standard screening practice.

Interestingly, most of the above studies focused on the cytokine profile in urine or peripheral blood from LN patients, and the results have often been inconsistent. Because specific organ or tissue involvement in SLE probably causes local cytokine aberrations that do not appear in systemic circulation, immunopathogenesis studies should focus on the specific sites of disease involvement. Lu et al. conducted a series of studies to investigate CXCL10 and CXCR3 mRNA expression specifically in the glomerulus and tubulointerstitium of patients with LN. Intrarenal CXCR3 expression was found to be significantly lower in LN patients than in controls but was inversely correlated with the degree of proteinuria and renal function, suggesting that the CXCR3 pathway is important in determining clinical severity rather than the histological pattern of LN [87]. Subsequently, they analyzed gene expression profiles of SLE patients with different histological types of LN who underwent repeated renal biopsies and found that tubulointerstitial CXCL10 expression decreased when the disease changed from proliferative or mixed nephritis to membranous nephropathy and correlated with the histological activity index; however, there was no difference in glomerular and tubulointerstitial CXCL10 expression between LN patients and controls [88]. These findings seem contradictory to those of the study by Enghard et al., which showed an enrichment of CXCR3^+^CD4^+^ T cells in inflamed kidneys of LN patients [82]. Moreover, previous immunohistochemistry and real-time RT-PCR analyses of renal biopsies from patients with LN (*n* = 19) showed that CXCR3-positive cells mainly overlapped with T cells infiltrating the tubulointerstitial compartment and were rarely found in glomeruli, which supported the results reported by Segerer et al. [35]. Nevertheless, we must note that the latter two studies lacked a comparison to healthy controls and could not explain the changes in CXCL10 expression caused by lupus kidney disease. Clearly, intrarenal CXCL10 and CXCR3 expression must be examined repeatedly, and it can be assumed that increased circulating levels of the chemokine CXCL10 and its receptor CXCR3 in LN patients are mainly produced by organs other than the kidney.

In addition, the diagnostic value of CXCL10 or CXCR3 in lupus nephritis was proposed in animal experiments. CXCR3^−/−^ MRL/lpr mice showed amelioration of nephritis with reduced glomerular tissue damage and decreased albuminuria, which may be owed to impaired trafficking of effector T cells to injured kidney [89]. Another study found that CXCR3^−/−^ NZB/NZW mice exhibited reduced production of total and anti-dsDNA antibodies of the IgG1 subclass but had normal titers of IgG2a and IgG2b antibodies compared to CXCR3^+/+^ NZB/NZW mice, indicating that therapeutic CXCR3 blockade could be beneficial for only a subgroup of patients with SLE [90]. Moreover, a recent research reported that CXCR3-deficient mesenchymal stem cells (MSCs) showed less infiltration into the nephritic kidney, less conjugation with endothelial cells, and weaker MMP-9 expression of MRL. Fas^lpr^ mice suggested that upregulation of CXCR3 in MSCs will be an effective strategy to improve therapeutic outcomes in SLE by increasing MSC infiltration into the kidney [91].

### 4.4. Renal Transplantation Rejection

There is a rapidly developing body of literature on the association between CXCL10 levels and inflammatory/immune processes occurring during organ transplantation. Several independent experimental and clinical investigations have documented and confirmed the important role of intragraft CXCL10 expression in the pathogenesis of graft failure owing to rejection in the kidney [14, 92].

To develop a noninvasive strategy for diagnosing rejection after renal transplantation, the patterns of intrarenal CXCL10 expression were evaluated in several different cohorts of patients and correlated with clinical and laboratory findings. Segerer et al. reported increased CXCL10 mRNA expression in transplant nephrectomy specimens from patients with acute rejection (AR) by RNase protection assay, supporting the importance of a Th1-type immune response during human renal allograft rejection [92]. A 3-month kidney graft biopsy protocol involving 257 patients confirmed higher CXCL10 mRNA expression in subclinical rejection, including borderline changes. In addition, higher intrarenal CXCL10 expression implied an increased risk of renal graft failure within one year after transplantation [93]. Another study of several inflammatory chemokine transcripts in renal allograft biopsies showed that both CXCL10 and CXCR3 expression levels were significantly elevated in allografts with subclinical or clinical AR. Thus, while allografts with subclinical AR maintain clinical function comparable to stable allografts, there transcriptional profile is consistent with that of allografts undergoing clinical AR [94]. CXCL10 is also required for graft failure owing to chronic and allograft nephropathy (CAN). CXCL10 expression was detected not only at the level of infiltrating inflammatory cells but also at the level of vascular, tubular, and glomerular structures in biopsy specimens from patients with CAN [95].

Enhanced CXCL10 production either in the graft or circulation of organ recipients is associated with its increased concentration in biological fluids (i.e., serum and urine). Rotondi et al. [96] ascertained the pretransplantation serum CXCL10 level in 316 cadaver kidney graft recipients and observed increased serum CXCL10 levels in patients who experienced graft failure, which supports the hypothesis that high pretransplant CXCL10 serum levels represent an important predictor of the risk of kidney graft rejection and transplant failure. In addition, multivariate analysis demonstrated that among the analyzed variables, CXCL10 had the highest predictive power for graft loss but not for death; in contrast, serum c reactive protein (CRP) levels correlated with death but not graft loss. In addition, high serum CXCL10 concentrations have been detected using luminex analysis during AR episodes [97]. Gene expression analysis of several chemokines and receptors in peripheral blood mononuclear cells (PBMCs) during acute renal allograft rejection also showed that CXCL10 expression was highly upregulated, especially in individuals with a poor response to antirejection therapy and a poor prognosis [98]. These results suggest that monitoring graft rejection following kidney transplantation based on CXCL10 serum levels or gene expression in peripheral blood is feasible.

The upregulation of urinary CXCL10 and its receptor CXCR3 has been previously described in patients experiencing biopsy-proven AR, including pediatric and adult renal allograft recipients [38, 99, 100]. Elevated urinary CXCL10 expression levels correlated with histological and clinical findings of rejection and were found to be more sensitive with greater predictive power than serum creatinine levels [38, 100, 101]. Subclinical tubulitis (SCT) has been associated with the later development of IFTA and diminished graft survival. Schaub et al. [102] investigated the correlation between CXCR3 chemokine levels and the extent of allograft inflammation to evaluate their potential use as an early detector of graft dysfunction. It was found that CXCL9 and CXCL10 urinary levels were significantly higher in the subclinical tubulitis Ia/Ib group than in the subclinical borderline tubulitis and the normal tubular histology groups, suggesting that CXCL9 and CXCL10 serve as urinary surrogates for the extent of subclinical graft inflammation. Ho et al. [103] further described a validation study to address the diagnostic value of urinary CXCL10 in tubulitis after renal allograft in another independent patient cohorts with a different subgroup. Although there were no significant differences in urinary CXCL10 concentration between borderline, subclinical, and clinical tubulitis groups, CXCL10 could accurately discriminate between tubulitis of any degree and normal renal transplant histology. Furthermore, the ratio of urinary CXCL10 to creatinine (CXCL10 : Cr) was found to distinguish borderline, subclinical, and clinical tubulitis from normal histology and IFTA. This study expands upon the previous finding of CXCL10 as a sensitive marker for graft inflammation and demonstrates that its elevation prior to elevated creatinine levels serves as an early marker of prognostic significance with regards to graft inflammation and long-term survival. In addition, a study of 213 consecutive patients who underwent 362 surveillance biopsies at 3 and 6 months confirmed that urinary CXCL10 levels correlate well with the extent of (sub)clinical tubulointerstitial inflammation [104]. Moreover, Matz et al. [101] documented that elevated urinary CXCL10 protein levels at early time points posttransplantation were predictive of graft function as assessed by GFR at 6 months, even in the absence of AR, suggesting that urinary CXCL10 might be a prognostic biomarker beyond allograft histology.

However, the clinical utility of CXCL10 is limited because it cannot distinguish BK nephropathy [100] or acute tubular necrosis from AR [67, 105]. Hricik et al. [106] proposed that urinary CXCL9, not CXCL10, was an excellent marker for excluding acute kidney allograft rejection and for stratifying patients in low- versus high-risk subsets for incipient allograft injury. Moreover, urinary CXCL10 does not sufficiently reflect inflammation in the vascular compartment [104]. Furthermore, it cannot predict AR in patients receiving alemtuzumab or basiliximab induction therapy, likely due to the depletion or inactivation of CXCR3+ responder cells [107]. Clearly, the clinical significance of such a noninvasive biomarker-driven biopsy strategy must be evaluated in a prospective study.

### 4.5. Renal Cell Carcinoma (RCC)

Recent data implicate CXCL10 in tumorigenesis mainly due to its antiangiogenic properties and promotion of immune activities. CXCL10 exerts angiostatic activity by inhibiting endothelial cell migration, proliferation, or differentiation [108–110]. It has been proven that CXCL10 could attenuate the process of new vessel formation and lead to reduced tumor growth in multiple human cancer models, such as non-small-cell lung cancer, colon–rectal cancer [111, 112]. In addition, data derived from studies in other human malignancies suggested that this antitumor effect is mainly through the regulation of immune response, currently available evidence indicates that (i) CXCL10 attracts CD8+ and CD4+ effector T cells to tumor sites and sites of inflammation [113], (ii) CXCL10 stimulates immune cells through Th1 polarization and activation [114, 115], (iii) CXCL10 are indispensable for robust responses to immune checkpoint inhibitors (anti-PD-1 and anti-CTLA-4) and improves therapeutic efficacy of radiotherapy [116, 117].

The importance of the CXCR3/CXCR3 ligand biological axis for inhibiting tumor growth has been substantiated in renal cell carcinoma (RCC). High-dose interleukin-2 produces durable, complete responses in a fraction of patients, providing proof of concept for the potential of immunotherapy in mRCC [118]. Several studies have shown that the expression level of CXCR3 and its ligands was significantly elevated in systemic high-dose interleukin-2 (IL-2) therapy, which suggests the promotion of tumor-specific immunity and association with prognosis [119–121]. Generally, it is accepted that CXCL10-based therapies for cancer due to its ability to attract CXCR3+ tumor-infiltrating lymphocytes (TILs) to tumor sites and sites of inflammation. However, recently, study about TILs in RCC demonstrated not only effector T cells but also regulatory T cells could be recruited via CXCR3 ligands, infiltration of Treg indicating suppression of effector T cells and poor prognosis of RCC patients [122–125]. In addition, systemic IL-2 treatment could augment the infiltration of CXCR3+ mononuclear cells and angiogenic ratio in human or murine RCC [120, 126]. CXCR3/CXCR3 ligand biologic axis induces the activation of immune response and closely related to prognosis. Recently, targeted agents including tyrosine–kinase inhibitors, vascular endothelial growth factor inhibitors, and mammalian target of rapamycin inhibitors have become available for treatment of mRCC [127–129]. It was observed that increased transcription for genes encoding CXCL9 and CXCL10 after treatment with nivolumab, an anti-PD-1 immune checkpoint inhibitor [130]. However, overexpression of these ligands could enhance RCC cell metastasis through regulation of adhesion, invasion, and migration phenotypes [131]. Thus, IFN-inducible CXCR3 ligands might affect tumor microenvironment via a paracrine manner and play a role in tumor progression and invasion via an autocrine manner [132].

### 4.6. Viral Infections

Viral infections often cause the onset or worsening of glomerulonephritis [133]. CXCL10 reportedly plays a prominent role during antiviral responses by binding to CXCR3 on Th1 lymphocytes and NK cells. Patients suffering from chronic hepatitis C have significantly elevated CXCL10 levels, and increased CXCL10 levels have been associated with AKI and glomerular pathologies of autoimmune origin [68, 134, 135].

Ample evidence confirms that treatment of cultured normal HMCs with polyinosinic-polycytidylic acid (poly IC) induces CXCL10 expression via the TLR3/IFN-*β*/MDA5 axis. Poly IC is a synthetic double-stranded RNA (dsRNA) analog that binds to TLR3 and is widely used to experimentally induce antiviral immune reactions due to its ability to induce the production of IFN-*β* and proinflammatory cytokines and the phosphorylation of signal transducer and activator of transcription 1 (STAT1), a key transcription factor in IFN signaling [133, 136–138]. Melanoma differentiation-associated gene 5 (MDA5), a member of the DExH box RNA helicase family, functions not only as a cytoplasmic receptor for double-stranded RNA [139] but also as a signaling molecule downstream of IFNs [140–142]. MDA5 is involved, at least in part, in poly IC-induced CXCL10 expression in MCs, while other MDA5-independent pathway(s) that mediate poly IC-induced CXCL10 expression require further study [143].

**Figure 1:**
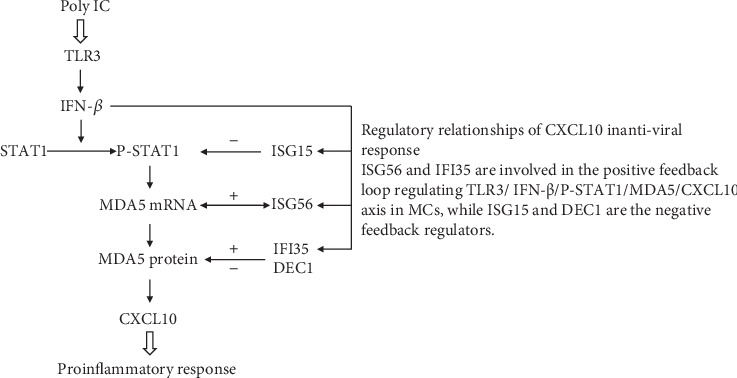
Regulatory relationships of CXCL10 in antiviral response. ISG56 and IFI35 are involved in the positive feedback loop regulating TLR3/IFN-*β*/P-STAT1/MDA5/CXCL10 axis in MCs, while ISG15 and DEC1 are the negative feedback regulators.

Moreover, Imaizumi et al. identified several IFN-stimulated genes (ISGs) that regulate CXCL10 expression in mesangial antiviral reactions and that are involved in GN associated with TLR3 activation. Interferon-stimulated gene 56 (ISG56) is induced by TLR3 signaling and positively regulates a downstream MDA5/CXCL10 proinflammatory signaling pathway [142]. A similar regulatory effect was observed for interferon-induced protein 35 (IFI35), an ISG that positively regulates the TLR3-dependent expression of CXCL10, at least in part, by enhancing MDA5 protein expression. However, IFI35 did not affect STAT1 phosphorylation [144]. In contrast, interferon-stimulated gene 15 (ISG15), especially unconjugated free ISG15, participates in a negative feedback loop in immune responses in MCs that decreases the phosphorylation of STAT1 and its downstream proteins [145]. Recently, another report showed that differentiated embryonic chondrocyte gene (DEC) 1, a basic helix-loop-helix (bHLH) transcription factor, was constitutively expressed in HMCs and served as an anti-inflammatory factor by inhibiting poly IC-induced MDA5/CXCL10 activation but not affecting STAT1 expression [138]. All these relationships are shown in Figure 1.

In addition to the essential roles of IFNs in regulating immune responses, TNF-*α* also induces CXCL10 in parallel by upregulating TNFR2 in human MC prestimulated with poly IC. This newly described TNFR2-mediated induction of CXCL10 is thought to be relevant for virus elimination in the early stage of infection downstream of the TLR3-dependent signaling cascade [146, 147].

## 5. Summary

Elevated CXCL10 and CXCR3 levels were detected in various renal diseases and correlated with disease progression. In addition, CXCL10 can be produced by renal resident cells such as mesangial cells, tubular epithelial cells, podocytes, and endothelial cells after stimulation in a cell type- and stimulus-specific manner. Studies have demonstrated that CXCL10 not only is responsible for renal inflammation by attracting T cells with a predominant Th1 profile but also can directly contribute to mesangial cell proliferation and migration, the maintenance of podocyte function, the inhibition of renal tubular epithelial cell proliferation, antitumor, and antiviral responses. Furthermore, CXCL10 has been identified as a biological indicator of disease severity and may be utilized as a prognostic indicator in AKI and LN (mainly described studies are summarized in Table 1). Nonetheless, the specific function of CXCL10 in different renal diseases is still unclear, and interactions between chemokines and specific cell types in the pathogenesis of various renal diseases must be investigated. Indeed, the chemokine CXCL10 may be a novel therapeutic target and potential biomarker for renal diseases.

## Figures and Tables

**Table 1a:** (a) Mesangial proliferative glomerulonephritis (MesPGN)

Function	Authors and year [Ref]	Studied subjects	Endpoints
CXCL10 promotes mesangial cell proliferation	Romagnani P et al. 1999 [30]	Patients/renal biopsies HMCs	High expression of CXCR3 by mesangial cells of patients with IgA nephropathy, membranoproliferative glomerulonephritis, or rapidly progressive glomerulonephritis. CXCL10 mediate both intracellular Ca^2+^ influx and HMC proliferation.
	Romagnani P et al. 2002 [33]	Patients/renal biopsies HMCs	Double-label immunohistochemical analysis for PCNA and *α*-SMA demonstrated that CXCL10 synthesis by resident glomerular cells was an extremely selective property of kidneys exhibiting PGN. The production of IP-10 and Mig by HMC can be downregulated by NO donors through cGMP-independent inhibition of NF-kappaB activation.
	Gao J et al. 2017 [55].	Cxcl10-deficient mice/ Habu nephritis model MMCs	Cxcl10^−/−^ mice exhibited mitigated mesangial proliferation via the ERK signaling pathway.
	Wörnle M et al. 2004 [54]	HMCs	CXCL10 induced HMC proliferation in a dose-dependent manner.

CXCL10 promotes mesangial cell apoptosis	Wu L et al. 2015 [27]	Mxi1-deficient mice/Habu nephritis model MMCs	CXCL10 expression induced by Mxi1 inactivation leads to mesangial cell apoptosis in murine Habu nephritis. CXCL10 expression was induced by TLR4–IRF3 signaling in Mxi1-inactivated mouse mesangial cells (MMCs).

CXCL10 maintains podocyte function	Han GD et al. 2003 [29]	Wistar rats/Thy1.1 GN mouse podocytes	The anti-IP-10 treatment given to the rats with Thy1.1 nephritis decreased the expression of nephrin and podocin, accompanied by exacerbated proteinuria, mesangiolysis, and matrix expansion.

**Table 1b:** (b) Acute kidney injury (AKI)

Function	Authors and year [Ref]	Studied subjects	Endpoints
CXCL10 as a potential injury biomarker of AKI	Ho J. et al. 2009 [67]	Patients/serum and urine	CXCL10 was upregulated postoperatively in patients with AKI, which corresponds temporally to the later phase(s) of tubular injury unique to patients with AKI.
	Vaidya V et al. 2008 [68]	Patients/urine	Urinary concentration of CXCL10 was significantly elevated in patients with clinically established AKI.
	E. Bautista et al. 2013 [69].	Patients/serum	Patients with influenza A/H1N1 infection and ARDS/AKI have an overproduction of IP-10 possibly contributing to kidney injury and are associated to a higher risk of death.

CXCR3 promotes recruitment of T cells	Fiorina P et al. 2006 [70]	CXCR3-deficient mice/renal I/R injury	CXCR3^−/−^ mice exhibited attenuated renal dysfunction and a lower percentage of CD4^+^IFN-gamma^+^ cells.
	Tsutahara K et al. 2012 [71].	Sprague-Dawley rats/renal I/R injury	The blocking of CXCR3 and CCR5 suppresses the infiltration of T lymphocytes.

CXCL10 inhibits renal tubular cell proliferation	Furuichi K et al. 2008 [39]	BALB/c mice/renal I/R injury Murine tubular epithelial cells	The numbers of Ki67-positive proliferating tubular cells were significantly increased in anti-CXCL10 Ab-treated mice after reperfusion.

**Table 1c:** (c) Lupus nephritis (LN)

Function	Authors and year [Ref]	Studied subjects	Endpoints
CXCL10 as an effective predictor of LN	Avihingsanon et al. 2006 [80].	Patients/urine	Urinary CXCL10 and CXCR3 levels could distinguish class IV LN from others with an accuracy greater than the current available clinical markers.
	Morimoto et al. 2009 [81].	Patients/serum	Increased serum CXCL10 level was found in patients with proliferative LN (World Health Organization (WHO) classes III and IV).
	Enghard P et al. 2009 [82].	Patients/renal biopsies, peripheral blood and urine	A selective enrichment of CXCR3^+^CD4^+^ T cells was found in the urine, and CXCR3 expression on urinary T cells correlated with disease activity as determined by the SLEDAI.
	Marie MA et al. 2014 [83].	Patients/urine	CXCL10 was considered a highly valid predictor of SLE nephritis with high sensitivity (100%) and specificity (98%).

Conflicting evidences on the reliability of CXCL10 as a diagnostic biomarker	Abujam B et al. 2013 [84].	Patients/serum and urine	Urinary CXCL10 was indicative of renal activity but was not better than conventional markers.
	El-Gohary A et al. 2016 [85].	Patients/serum and urine	No difference between LN and SLE patients without renal involvement nor between active renal SLE and active non-renal SLE patients.
	Watson L et al. 2012 [86].	Patients/urine	Urinary concentrations of CXCL10 could not distinguish active from non-active renal disease in patients with juvenile-onset systemic lupus erythematosus (JSLE).
	Lu J et al. 2011 [87].	Patients/renal biopsies	CXCR3 pathway is important in determining clinical severity rather than the histological pattern of LN. No difference in glomerular and tubulointerstitial CXCL10 expression between LN patients and controls.

CXCR3 mediates renal Th1 and Th17 immune response	Steinmetz OM et al. 2009 [89].	CXCR3^−/−^ MRL/lpr mice	CXCR3^−/−^ MRL/lpr mice showed amelioration of nephritis with reduced glomerular tissue damage and decreased albuminuria and T cell recruitment.

CXCR3 promotes the production of IgG1 autoantibodies	Moser K et al. 2012 [90].	CXCR3^−/−^ NZB/NZW mice	CXCR3 has an effect on (auto)antibody production but is not essential for lupus pathogenesis in NZB/NZW mice.

CXCR3 increases MSCs infiltration	Lee JH et al. 2018 [91].	MRL.Fas^lpr^ mice	CXCR3-deficient mesenchymal stem cells fail to infiltrate into the nephritic kidney and do not ameliorate lupus symptoms in MRL.Fas^lp^r mice

**Table 1d:** (d) Renal transplantation rejection

Function	Authors and year [Ref]	Studied subjects	Endpoints
Enhanced CXCL10 production in the graft of AR	Segerer S et al. 2001 [92].	Patients/renal biopsies	Increased CXCL10 mRNA expression in transplant nephrectomy specimens from patients with acute rejection (AR) by RNase protection assay.
	Matl I et al. 2010 [93].	Patients/renal biopsies	Higher CXCL10 mRNA expression in subclinical rejection, including borderline changes. Higher intrarenal CXCL10 expression implied an increased risk of renal graft failure within one year after transplantation.
	Lo DJ et al. 2011 [94].	Patients/renal biopsies	CXCL10 and CXCR3 expression levels were significantly elevated in allografts with subclinical or clinical AR.

Elevated serum CXCL10 expression in AR	Rotondi M et al. 2004 [96]	Patients/serum	High pretransplant CXCL10 serum levels represent an important predictor of the risk of kidney graft rejection and transplant failure.
	Huang H et al. 2014 [97].	Patients/serum	High serum CXCL10 concentrations have been detected using luminex analysis during AR episodes.
	Mao Y et al. 2011 [98].	Patients/serum	CXCL10 were highly up-regulated in peripheral blood mononuclear cells in acute rejection and poor response to anti-rejection therapy.

Urinary CXCL10 as a potential noninvasive biomarkers for AR	Schaub et al. 2009 [102]	Patients/urine	CXCL9/CXCL10 potential noninvasive biomarkers for subclinical tubulitis.
	Ho et al. 2011 [103]	Patients/urine	CXCL10 : Cr sensitivity of 73.3% and specificity of 72.7%.
	M. Matz et al. 2006 [101]	Patients/urine	Incidence of AR: Urinary IP-10 protein observed 2/3 d prior to biopsy with 71% sensitivity and 95% specificity. Long term graft function: Urinary IP-10 predictive of GFR at 6 month posttransplant.
	P. Hirt-Minkowski et al. 2012 [104]	Patients/urine	Urinary CXCL10 levels correlate with the extent of (sub) clinical tubulointerstitial inflammation.
	D. E. Hricik et al. 2013 [106]	Patients/urine	Urinary CXCL9 is a risk-stratifying biomarker for kidney transplant injury.
